# Plasma BNP level combined with surgical Apgar score to predict operative major cardiac adverse events in malignant obstructive jaundice patients

**DOI:** 10.12669/pjms.325.10302

**Published:** 2016

**Authors:** Wei Yu, Changshan Huang, Qian Wang, Erjiang Zhao, Yuechao Ding, Tao Huang, Chao Ma, Bo Meng

**Affiliations:** 1 Wei Yu, Department of Hepatobiliary & Pancreatic Surgery, Affiliated Tumor Hospital of Zhengzhou University, Zhengzhou 450008, China; 2 Changshan Huang, Department of Hepatobiliary & Pancreatic Surgery, Affiliated Tumor Hospital of Zhengzhou University, Zhengzhou 450008, China; 3 Qian Wang, Department of Hepatobiliary & Pancreatic Surgery, Affiliated Tumor Hospital of Zhengzhou University, Zhengzhou 450008, China; 4 Erjiang Zhao, Department of Hepatobiliary & Pancreatic Surgery, Affiliated Tumor Hospital of Zhengzhou University, Zhengzhou 450008, China; 5 Yuechao Ding, Department of Hepatobiliary & Pancreatic Surgery, Affiliated Tumor Hospital of Zhengzhou University, Zhengzhou 450008, China; 6 Tao Huang, Department of Hepatobiliary & Pancreatic Surgery, Affiliated Tumor Hospital of Zhengzhou University, Zhengzhou 450008, China; 7 Chao Ma, Department of Hepatobiliary & Pancreatic Surgery, Affiliated Tumor Hospital of Zhengzhou University, Zhengzhou 450008, China; 8 Bo Meng, Department of Hepatobiliary & Pancreatic Surgery, Affiliated Tumor Hospital of Zhengzhou University, Zhengzhou 450008, China

**Keywords:** Obstructive jaundice (OJ), Main adverse cardiac events (MACE), Plasma brain natriuretic peptide (BNP), Surgical Apgar score

## Abstract

**Objective:**

To investigate the predictive effect of major adverse cardiac events (MACE) in malignant obstructive jaundice (OJ) patients using plasma brain natriuretic peptide (BNP) level and surgical Apgar scoring (SAS) system.

**Methods:**

Forty one malignant OJ patients undergoing surgical treatments were studied at a single center. Pre-and postoperative plasma BNP level, total bilirubin (TBil) and data of cardiac function (HR, CVP, CI, LVEF%) were detected, the SAS was calculated during the surgery, the relationship of both plasma BNP level and SAS with MACE after surgery was analyzed.

**Results:**

Thirteen patients out of 41 (31.71%) experienced MACE without cardiac death. OJ patients had a higher plasma BNP level than baseline before operation (191.61±105.76 pg/ml VS 175 pg/ml, P<0.05), the cardiac function data was improved (CVP: t=4.761, *p*=0.000; CI: t=3.539, *p*=0.001; LVEF%: t=3.632, *p*=0.001) after the operation. Patients with lower SAS had increasing incidence of MACE after surgery.

**Conclusion:**

Malignant OJ patients with higher preoperative BNP level and lower surgical Apgar score were identified at high risk of MACE after surgery.

## INTRODUCTION

Obstructive jaundice (OJ) is a common disease worldwide due to tumor, inflammatory and lithiasis of the bile duct tree and the pancreas. OJ can induce people into systemic pathologic alteration, such as respiratory inhibition, renal failure, coagulopathy and cardiac disfunction.^1^-^4^ Surgery is considered to be the most available treatment of OJ with a postoperative morbidity up to about 20-30%.^5^ Main adverse cardiac events (MACE) is one of the most common postoperative complications in OJ patients.

Cardiac disfunction usually plays an critic role of postoperative MACE which leads to shock, hypotension and even death.^6^ Thus, to detect and intervene in cardiac disfunction before operation become very important for people with OJ. Unfortunately, normal electrocardiogram (ECG) or Holter examination appeared to be hysteretic to a great extent, since most cardiac injury induced by OB affects functional level rather than abnormal mechanical and electrical activities.

Plasma brain natriuretic peptide (BNP), a sensitive biomarker of cardiocyte in response to stretch which can be excreted to cardiac ventricles initially, has been proved to be valuable for the diagnosis and therapy guidance for most heart diseases.^7^,^8^ Recent studies have also demonstrated that plasma BNP level is closely associated with postoperative cardiovascular adverse event in non-cardiac surgery.^9^ Plasma BNP level has also been recognized as a diagnostic marker of left ventricular disfunction.^10^ Several studies show that OJ patients have a higher baseline of BNP than normal people,^11^ even if there is no cardiac abnormality detected. Also researchers found that plasma BNP level significantly decreased accompanied with better cardiac function after the internal biliary drainage procedure carried out.^12^ However, the relationship of plasma BNP level and postoperative MACE in OJ patients remains unclear.

It is widely accepted that changes of vital signs during the operation can also affect the postoperative outcomes. But precise assessment system is rarely reported. The SAS, a ten- point scoring system based on lowest intraoperative mean arterial blood pressure (MAP), lowest heart rate (HR), and estimated blood loss (EBL) during operation has proven to be effective in identifying patients at high risk for postoperative complications in various subspecialties.^13^-^16^ The SAS was inversely associated with the postoperative mortality and morbidity, that means patients with higher score has less postoperative complications and better outcomes.^17^,^18^ The aim of this study was to investigate the relationship of postoperative MACE and preoperative plasma BNP level as well as SAS in OJ patients.

## METHODS

### Subjects

Data of 41 OJ patients who received surgical treatment in our hospital (January 2012-July 2014) were collected. General characteristics are shown in Table-I. The diagnose of OJ was according to case history, physical signs, laboratory examination and image technique. Patients with heart disease, pulmonary hypertension, chronic obstructive pulmonary disease and chronic kidney disease were excluded from this cohort in order to eliminate the impact of the BNP base level.

**Table-I:** 10-point Surgical Apgar score system.

*Items*	*0 point*	*1 point*	*2 points*	*3 points*	*4 points*
EBL (ml)	>1000	601-1000	101-600	≤100	----
lowest MAP (mmHg)	<40	40-54	55-69	≥70	----
Lowest HR (beast/min)	>85	76-85	66-75	56-65	≤55

### Operation Details

All patients received surgical treatment under general anesthesia, including pancreaticoduodenectomy in 17 cases, hilarcholangiocarcinoma radical resection in 14 cases (combined with right hemihepatectomy in 5 cases; with the left hemihepatectomy in 4 cases; with hepatic segment resection in 5 cases), gallbladder carcinoma radical resection in 5 cases, cholangiojejunostomy in 5 cases, no patient died during the operation. The volume of postoperative rehydration fluids was 40-60 ml/Kg during the fasting time.

### BNP Detection

Blood samples were collected with chilled tubes containing ethylenediaminetetraacetic acid and aprotinin 1 d before and 3 d after the operation through the peripheral vein. The plasma was seperated at 3000 rpm for 15 minutes, plasma BNP level was detected using a fluorescent immunochromatographic assay (BGHG-Triage, USA) with a normal value of 100 pg/ml or less. Meanwhile the liver function data was determined with the routine protocol in our hospital.

### SAS Calculation

The radial artery puncture was carried out 30 minutes before the operation for real-time monitoring of the mean arterial blood pressure and the heart rate. Estimated blood lose of the surgery was counted based on the negative pressure aspirated volume and the number of wet packs (50 ml per piece). The SAS was immediately calculated after the operation. Table-II.

**Table-II:** General characteristics of 41 patients.

*Characteristic*	
Age (year)	59.69+9.62
*Sex*	
Male	29
female	12
*Cause of obstruction*	
PC	9
BDC	23
GC	5
PAC	4
EBL (ml)	347.55±133.82
Lowest MAP (mmHg)	76.39±34.27
Lowest HR (beats/min)	65.54±24.91
Albumin (g/L)	38.77±7.29
Tbil (μmol/L)	239.88±92.48
AST (U/L)	121.56±43.13
PT (S)	15.31±3.47
BNP (pg/ml)	191.61±105.76

*Abbreviation:* PC: Pancreatic carcinoma; BDC: bile duct carcinoma; GC: gallbladder carcinoma; PAC: periampullary carcinoma; EBL: estimated blood loss; MAP: mean arterial blood pressure; HR: heart rate; ALT: alanine transaminase; PT: Prothrombin Time.

### Cardiac function and MACE

The data of the cardiac function including heart rate (HR, beats/min), central venous pressure (CVP, cmH_2_O), cardiac index (CI, L/m^2^·min) and left ventricular ejection fraction percentage (LEVF%) were detected one day before the operation (GE LOGIQ E8, USA), the postoperative data were detected with bedside color Doppler ultrasonography (GE LOGIQ E, USA) three days after the operation. Before the examination all of the participants were asked to lay still at least for 15 minutes. Postoperative MACE contains the performance of heart failure, cardiac insufficiency, cardiac asthma, severe arrhythmia, myocardial infarction and cardiac death.

### Statistical Analysis

Statistical analysis was performed using SPSS17.0 statistics software. Results were expressed as mean ± SD or percentage. Chi-square test was used for percentage comparisons and paired t test was used for mean comparison. *p*<0.05 indicated that the difference had statistical significance.

## RESULTS

### Postoperative MACE

Thirteen patients in 41 (31.71%) developed of MACE, which consisted of three cases of heart failure, three cases of cardiac insufficiency, one case of cardiac asthma, two cases of severe arrhythmia, two cases of myocardial infarction, no cardiac death.

### Changes of plasma BNP level, total bilirubin concentrations and cardiac function

OJ patients have an higher preoperative level of plasma BNP than the baseline on average. The average concentrations of total bilirubin was significant decreased after the operation (t=13.351, *p*=0.000), concomitant decrease of the plasma BNP level (t=4.784, p=0.000) was also observed. The postoperative cardiac function was improved (CVP: t=4.761, *p*=0.000; CI: t=3.539, *p*=0.001; LVEF%: t=3.632, *p*=0.001) except the data of HR (t= -0.572, *p*=0.570) (Table-III).

**Table-III:** Pre- and postoperative changes of TBil, BNP and cardiac function.

*Items*	*Preoperative-postoperative*	*t*	*P value*
TBIL	71.756 ±34.414	13.351	0.000
BNP	31.366± 41.980	4.784	0.000
HR	-1.220 ±13.650	-0.572	0.570
CVP	1.683 ± 2.263	4.761	0.000
CI	-0.168 ±0.304	-3.539	0.001
LVEF	-1.707±3.010	-3.632	0.001

### Relationship of preoperative plasma BNP level, SAS and MACE

**Fig.1:**
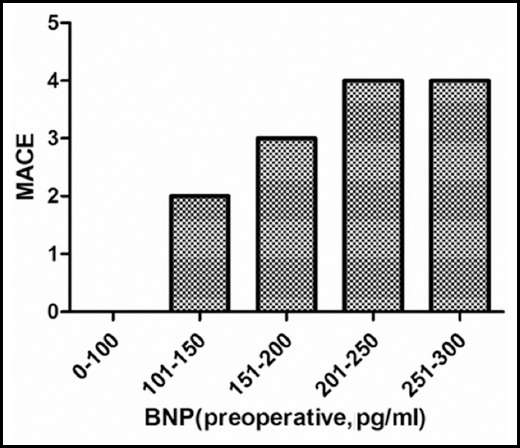
Distribution of MACE according to preoperatoive plasma BNP level.

**Fig.2:**
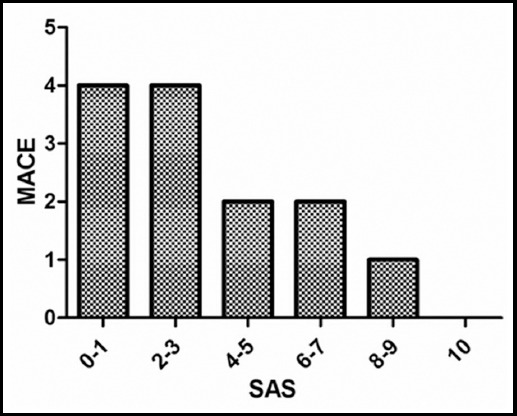
Distribution of MACE according to SAS.

**Fig.3:**
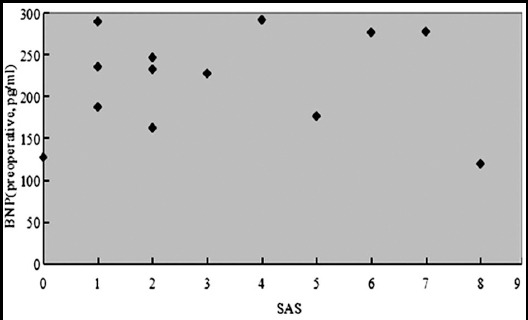
Distribution of patients developed of MACE according to preoperative BNP level and SAS.

The postoperative MACE was significantly associated with the preoperative plasma BNP level. Patients who developed of MACE have a higher preoperative plasma BNP level than that without MACE. Eight cases of MACE developed with a preoperative plasma BNP level rank from 200 to 300 pg/ml, only one case of MACE developed with a preoperative plasma BNP level less than 150pg/ml (Fig.1). Also, Patients who developed MACE has a lower SAS than that without MACE. Among the total 13 cases of MACE, 4 cases developed with a score from 0 to 1, 4 cases developed with a score from 2 to 3; only 1 case developed MACE with a score more than 7 (Fig.2). Most MACE has been developed in patents with higher preoperative plasma BNP level and lower SAS (Fig.3).

**Table-IV:** Relationship of postoperative MACE and preoperative plasma BNP level and SAS.

*Items*	*MACE*	*No-MACE*	*t*	*P value*
TBIL	223.85 ± 86.916	247.32 ± 95.570	-0.752	0.456
BNP	269.23 ± 112.158	155.57 ± 82.048	3.667	0.001
SAS	3.38 ± 2.501	6.82 ± 2.639	-3.942	0.000

## DISCUSSION

Low or medium level of serum bilirubin has been proved to acts as protective effect for cardiovascular system through antioxygen free radical activity in non-jaundice people.^19^-^21^ Based on these facts, some researchers even add small dose of bilirubin to alleviate the risks of cardiovascular and other systems.^22^ In OJ patients, the matter turns out completely opposite. Since Green et al. described the cardiac injury of OJ firstly in 1986,^23^ studies afterwards have continuously confirmed that cardiac disfunction is one of the common complications in patients with OJ.^24^ Experimental studies indicated that effects of bile duct ligation on myocytes including decrease of contraction rates and amplitude leads to cardiac disfunction potentially.^25^ It is quite necessary to predict and intervene of MACE in the early stage of OJ operations.

BNP has been verified as an objective indicators in extend and severity of heart failure, cardiac infarction and acute coronary syndrome,^26^-^28^ In cardiac surgery, plasma BNP level is associated with postoperative mortality as well as a strong prognostic factors of long-time outcomes.^29^ Dernellis et al. documented that preoperative plasma BNP concentration is an independent predictor of postoperative cardiac events in non-cardiac surgeries, patients were identified at high risk when BNP is more than 189 pg/ml.^30^ In another associated meta analysis of non-cardiac surgery, high concentrations of plasma BNP remains an independent factor and predictor of short- and long-term MACE.^31^ In previous studies a higher level of plasma BNP in OB patients was compared with normal people. It showed significant reduction after the effective treatment of OB.^12^ Not only the similar results was found in current study, but also the association of preoperative plasma BNP level and postoperative MACE was detected.

Because of the systemic physical injury of OJ, most patients performed too bad to tolerate the operation. Since lack of the description for accurate assessment of preoperative situations, it is very important to evaluate the intraoperative performance for identifying patients most at risk of developing major complications. Recent study showed that serum bilirubin level ≥300 μmol/L increase early stage morbidity and decreases the long-term survival in patients underwent pancreaticoduodenectomy.^32^ The heart play as a central role to maintain intraoperative circulation stabilization, and acts more likely to be affected by the circulation disorders. The SAS which developed based on three simple data of the circulation system, although mostly are transient, is still supposed to be an predictor for postoperative MACE. Since the SAS is found to be a useful tool to evaluate the relationship between the intraoperative performance and postoperative complications,^33^ special attention should be paid to lower score patients. In this study, we found that most MACE developed in patients with a score less than 4, only one patient developed MACE when the score is more than 7.

In this study, we found that high level of preoperative plasma BNP level with a lower SAS increase the risk of postoperative MACE in OJ patients. As described above, higher plasma BNP level indicate more severe preoperative cardiac injury, while lower SAS means worse intraoperative performance. Even the total bilirubin was decreased after the operation, the volatility of intraoperative vital signs also leads to a “second attract” to the heart. According to this understanding, maybe the preoperative biliary drainage should be reconsidered.

### Limitations of the study

First, we did not detect the estimated glomerular filtration rate (eGFR) when patients show normal renal function before the operation, since the BNP level is affected by the eGFR significantly.^34^ Second, all the data and the subjects were collected in a single center, the error range has not been limited to a smallest extend compared with multi-center studies. Third, the postoperative cardiac function was evaluated based on the bedside data, we cannot eliminate the error compared with normal ultrasonic data. Forth, all the data in this study were obtained from patients with malignant tumor, large sample studies that contains non-cancer cases is needed. Finally, routine use of drugs after the operation could also affect the plasma BNP level and cardiac function such as furosemide and triphosadenine.

In conclusion, this study suggests that high level of total bilirubin contribute to cardiac injury and postoperative MACE in OJ patients. Combined plasma BNP level detection with SAS provide an feasible method in identifying patients of developing postoperative MACE. Because of the limitations, further research is needed before the method is more widely applied.
